# Isolation of a highly virulent colibactin-positive tumor-promoting strain of *Escherichia coli* from the gut microbiota of an adult

**DOI:** 10.1128/msphere.00219-26

**Published:** 2026-05-07

**Authors:** Allison M. Weis, O'Connor J. Matthews, Rickesha Bell, Nicole Lynn Pershing, Annika Dankwardt, Brittany A. Fleming, Biljana Gigic, Martin Schneider, Sheetal Hardikar, Adetunji T. Toriola, David Shibata, Christopher I. Li, Doratha A. Byrd, W. Zac Stephens, Cornelia M. Ulrich, Matthew A. Mulvey, June L. Round

**Affiliations:** 1 Department of Pathology, Division of Microbiology and Immunology, University of Utah School of Medicine, Huntsman Cancer Institute 12348 , Salt Lake City, Utah, USA; 2 Division of Gastroenterology, Hepatology, and Nutrition, Department of Internal Medicine, University of Utah School of Medicine 12348 , Salt Lake City, Utah, USA; 3 School of Biological Sciences, University of Utah, Salt Lake City, Utah, USA; 4 Henry Eyring Center for Cell & Genome Science, University of Utah, Salt Lake City, Utah, USA; 5 Office of Comparative Medicine, University of Utah School of Medicine 12348 , Salt Lake City, Utah, USA; 6 Department of Pediatrics, Division of Pediatric Infectious Diseases, University of Utah School of Medicine 12348 , Salt Lake City, Utah, USA; 7 Department of General, Visceral and Transplantation Surgery, Heidelberg University Hospital, Heidelberg, Germany; 8 Clinic for General, Visceral, Thoracic, Transplantation, and Pediatric Surgery, University Hospital Gießen and Marburg GmbH, Gießen, Germany; 9 Department of Population Health Science, University of Utah School of Medicine, Huntsman Cancer Institute 12348 , Salt Lake City, Utah, USA; 10 Division of Public Health Sciences, Department of Surgery, Siteman Cancer Center Washington University School of Medicine, St. Louis, Missouri, USA; 11 Department of Surgery, University of Tennessee Health Science Center, Memphis, Tennessee, USA; 12 Divison of Public Health, Fred Hutchinson Cancer Center 7286, Seattle, Washington, USA; 13 Department of Cancer Epidemiology, H. Lee Moffitt Cancer Center & Research Institute, Tampa, Florida, USA; University of California Davis, Davis, California, USA

**Keywords:** human microbiome, *E. coli*, pathogen, colon cancer, tumor promoting microbes

## Abstract

Recent studies have pointed to critical roles for microbes in both exacerbation of and protection from the development of colon cancer. While much has been learned, the field remains understudied, with functional studies available for only a handful of bacteria. To identify novel microbes associated with colorectal cancer (CRC) development, we employed a preclinical chemical carcinogenesis CRC mouse model using germ-free mice that were colonized with human microbiotas. During the course of these studies, we identified a microbiota that exacerbated CRC, from which we isolated an *Escherichia coli* strain that had disseminated to the mouse kidneys. This strain, which we designated as AW001, was genetically similar to the reference adherent-invasive *E. coli* (AIEC) strain NC101 and encoded the DNA-damaging toxin colibactin. In relevant animal models, AW001 worsened both colitis and sepsis, making it a colitogenic AIEC-like strain with the capacity to cause invasive systemic infections similar to extraintestinal pathogenic *E. coli* (ExPEC). This strain will be a relevant tool to study human-associated intestinal *E. coli* strains capable of causing disease in mice.

**IMPORTANCE:**

Colorectal cancer (CRC) is a significant burden on human health. A growing body of work has pointed to critical roles for microbes in the exacerbation of and protection from the development of CRC. Specific *Escherichia coli* strains can produce colibactin, a genotoxin that has been implicated in exacerbating CRC. In this study, we tested human microbiotas in a mouse model of CRC and isolated a colibactin*-*positive *Escherichia* coli strain that led to tumorigenesis, disseminated from the gut to the mouse kidneys, caused death, and worsened both colitis and sepsis in murine models. Identification of this strain enhances our collective knowledge and adds an important tool for future studies on the role of microbes and CRC tumorigenesis.

## INTRODUCTION

Globally, there are 1.9 million new cases of colorectal cancer (CRC) every year, with rates predicted to double by 2040 (1, 2). In the United States, CRC is the second most common cause of cancer death (3), and there has been an emergence of cases of CRC in younger individuals without clear causes or genetic predisposition (3, 4). Microbes have been implicated in ~20% of all human cancers (5), and recent clinical and epidemiological studies have shown strong associations of CRC with bacteria residing in the gut (6–8). Indeed, emerging research has pointed to a myriad of roles for microbes in both exacerbation of and protection from the development of CRC (9). While much has been learned, the field remains understudied, with mechanistic insight available for only a handful of bacteria.

*Escherichia coli* strains are common gut commensals, but subsets of these bacteria can also act as devastating and persistent pathogens. Pathogenic strains of *E. coli* can cause many types of infections, including diarrhea, urinary tract infections, neonatal meningitis, bacteremia, and sepsis (10–12). Further, *E. coli* strains that carry the *pks*+ gene cluster, which encode a secreted DNA-damaging factor known as colibactin, can promote tumor development and are associated with increased rates of CRC (13–15). In this study, we colonized germ-free mice with human microbiotas from patients with CRC and age-matched asymptomatic controls and assessed *de novo* tumorigenesis in a preclinical chemical carcinogenesis CRC mouse model to identify novel microbes that could modify CRC development. Surprisingly, during the course of these studies, we isolated from the kidneys of a control mouse inoculated with a non-CRC human microbiota a novel invasive, colitogenic *E. coli* harboring the colibactin gene. Our findings highlight the complex interactions between microbial virulence factors and the mammalian host dictating disease risk.

## RESULTS

### The microbiota from an individual without colon cancer exacerbated tumorigenesis and death in a preclinical CRC mouse model

**Fig 1:**
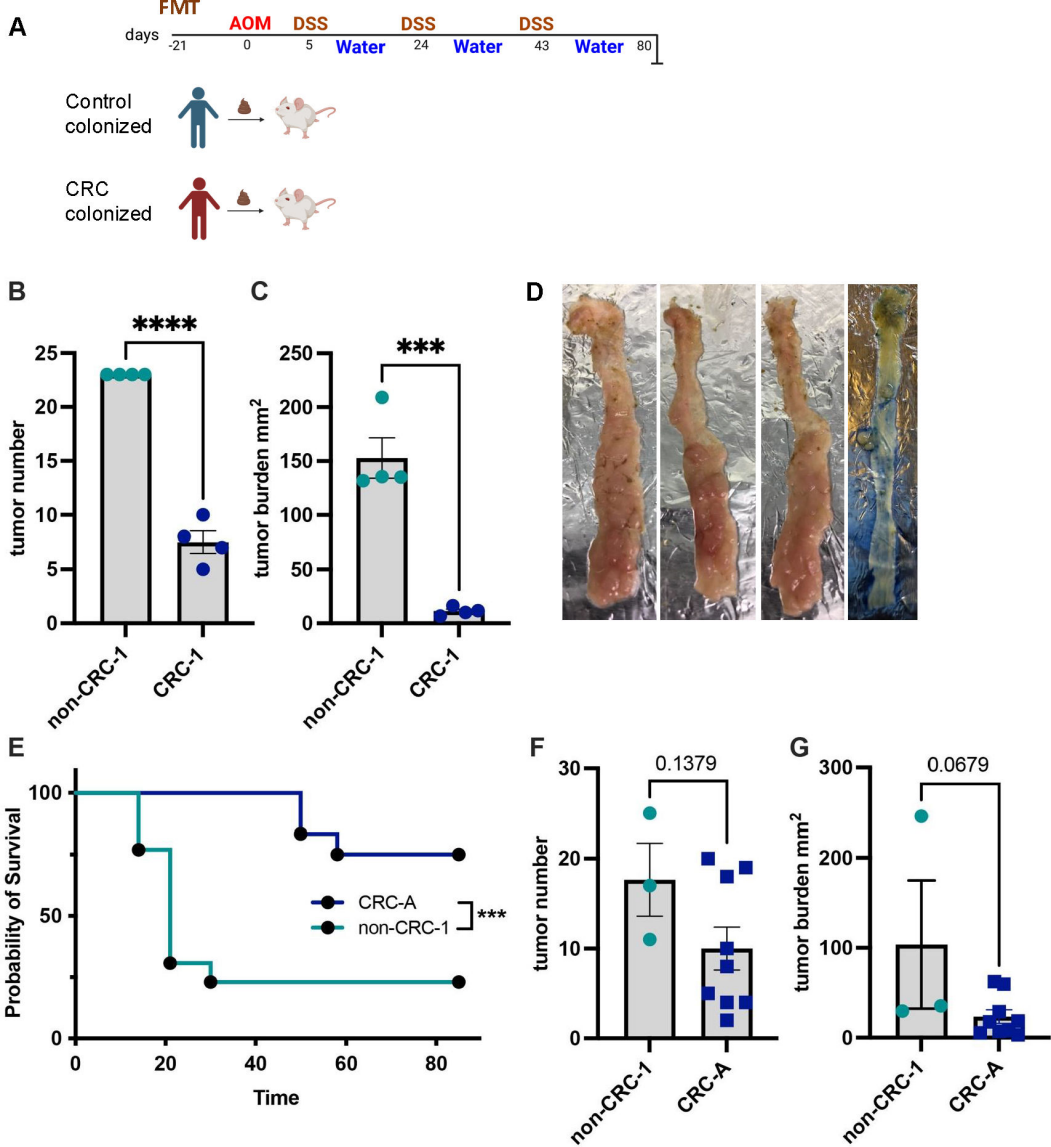
The microbiota from an individual without colon cancer exacerbated tumorigenesis and death in a preclinical CRC mouse model. (**A**) Schematic of the AOM/DSS CRC mouse model, which relies on a single injection of AOM and three subsequent DSS pulses with water cycles in between. Ex-GF Swiss Webster mice after orally gavaged and engrafted microbiotas at the end of the AOM/DSS model (**B–D**, *N* = 8). (**B**) Tumor number and (**C**) tumor burden plotted as the sum of tumor sizes in mm^2^ between non-CRC-1 human microbiota and CRC-1 microbiota. (**D**) Images of three of the colons from non-CRC-1 from this experiment are shown on the left. The rightmost colon is representative of a typical mouse colon from the AOM/DSS model with several tumors among mostly normal tissue. (**E–G**) AOM/DSS results from *F1* Swiss Webster progeny from non-CRC-1 (*N* = 12) and CRC-A (*N* = 13). (**E**) Kaplan–Meier survival plot comparing non-CRC-1 and CRC-A. *P* = 0.0022, by log-rank Mantel–Cox test, and *P* = 0.0009 by Gehan-Breslow-Wilcoxon test between non-CRC-1 and CRC-A. (**F**) Tumor number and (**G**) tumor burden between groups. Differences were analyzed using unpaired two-tailed *t*-tests: ns. *P* ≥ 0.05; *, *P* ≤ 0.05; **, *P* ≤ 0.005; ***. *P* ≤ 0.0005; ****, *P* ≤ 0.0001. Error bars are ± SEM. All mouse groups reflect pooled data from both sexes, which represented half of the subjects in each group.

To identify microbes of relevance to the development of human CRC, we utilized a well-established CRC murine model, the AOM-DSS model (16), to compare the effects of the fecal microbiota from a patient with stage 2 colon cancer (microbiota CRC-1) to those of a control microbiota from a disease-free, asymptomatic, age- and sex-matched individual (non-CRC-1). Stool samples from these individuals were collected in collaboration with the ColoCare Study, an international cohort of newly diagnosed stage I–IV colorectal cancer patients (17, 18), and used to inoculate adult germ-free mice via oral gavage, as previously described (19, 20). Following inoculation, mice were housed with minimal disruption for 21 days to allow the microbial communities time to stabilize. The animals were injected once with the carcinogen azoxymethane (AOM), followed by delivery of three pulses of dextran sodium sulfate (DSS) (Fig. 1A). These treatments trigger *de novo* tumorigenesis that can be quantified grossly after a period of 80 days (16). We anticipated that transfer of the control microbiota non-CRC-1 would result in lower, or similar, numbers of tumors relative to the CRC-1 microbiota based on our previous studies (21). However, following the AOM/DSS treatments, animals containing the non-CRC-1 microbiota had significantly higher tumor numbers and total tumor burden within each mouse colon compared to mice inoculated with the matched CRC-1 microbiota (Fig. 1B and C). Indeed, each colon from the control group contained a nearly confluent field of tumors covering most of the colon surface (Fig. 1D, left three colon images), compared to a typical mouse colon after the AOM/DSS model (Fig. 1D, right most colon image).

To further assess the effects of the non-CRC-1 microbiota, the F1 progeny of wild-type (WT) mice carrying the non-CRC-1 microbiota were compared in the AOM/DSS CRC model with F1 mice from breeders containing a pro-tumorigenic microbiota (CRC-A) obtained from a patient with stage IV CRC. Surprisingly, in these experiments, mortality following the AOM/DSS treatments was very high, particularly among mice with the non-CRC-1 microbiota. In fact, 9 out of 12 mice from the non-CRC-1 microbiota group died compared with 3 out of 12 mice containing the CRC-A microbiota (Fig. 1E). Indeed, mice in the non-CRC-1 group started dying even prior to completion of the AOM/DSS treatments, with three succumbing after the first DSS dose and six more dying on day 21, just prior to the second DSS treatment. At these early time points, DSS treatment has already perturbed the gut barrier, but mortality is rarely observed. The non-CRC-1 mice that did survive the AOM/DSS treatments were analyzed for their tumor number and burden compared with the CRC-A mice, most of which were still alive. Mirroring results obtained with the CRC-1 mice (see Fig. 1B and C), the surviving non-CRC-1 F1 mice tended to have increased tumor numbers and an overall elevated cumulative tumor burden relative to the CRC-A mice, although these differences were not significantly different due to the low survival rate of the non-CRC-1 F1 mice (Fig. 1F and G).

Next, we used PCR to probe the non-CRC-1, CRC-1, and CRC-A microbial communities from the original human inoculum for the presence of specific bacterial toxin genes and species that were previously shown to promote the development of CRC. These included genes for the *E. coli*-associated genotoxin colibactin subunits (*clbA*, *clbP*, *clbB*) (13, 22), the *Bacteroides fragilis* toxins BFT-1 and BFT-2 (13), and the bacterium *Fusobacterium nucleatum* (23). We found that only the non-CRC-1 community contained the colibactin subunit genes *clbA*, *clbP*, and *clbB*, as well as genes for BFT-1 and BFT-2 (Table 1). In contrast, neither CRC-1 nor CRC-A contained the colibactin subunit genes, but BFT-1 was detected in CRC-1. All three microbiotas contained *F. nucleatum*, as assessed using species-specific 16S rDNA primers. In addition, we confirmed that *E. coli* and the colibactin toxins were also present by PCR in the mouse fecal samples from non-CRC-1 in the experiments in Fig. 1 (Table S1). Taken together, our data show that all three microbiotas from individuals with or without CRC contain microbes and microbial toxins known to exacerbate CRC; however, the non-CRC-1 microbiota promoted more tumors in mice.

**TABLE 1:** PCR of known bacterial toxins associated with CRC from human microbiotas^*a*^

Microbiota	*E. coli* colibactin toxin			*B. fragilis* toxin		*F. nucleatum*
	ClbA	ClbP	ClbB	BFT-1	BFT-2	Fn
CRC-1	–	–	–	+	–	+
Non-CRC-1	+	+	+	+	+	+
CRC-A	**–**	–	–	–	–	+

^*a*^ +, present; –, absent.

### *E. coli* isolation and comparative genomics

**Fig 2:**
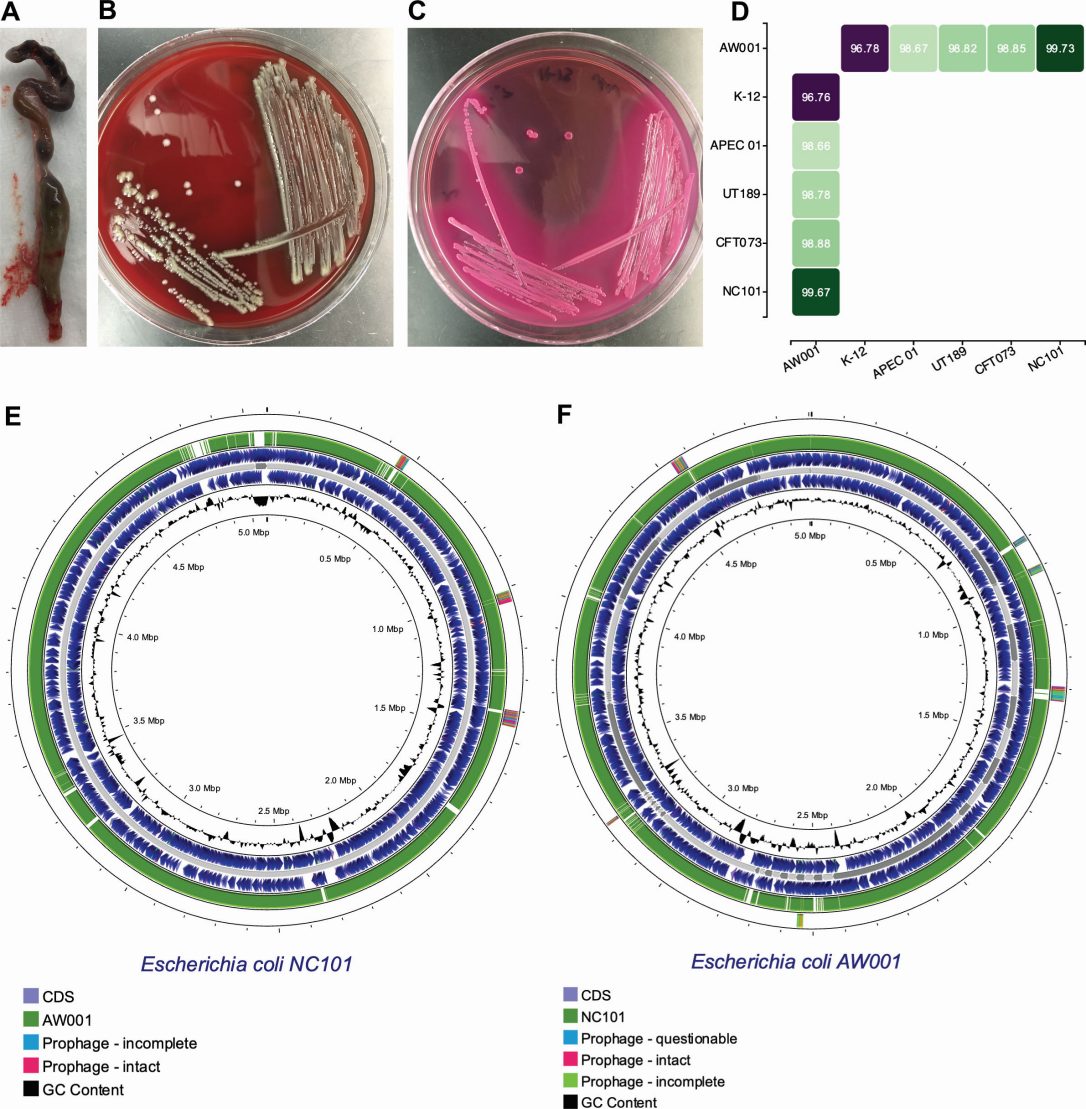
*E. coli* isolation and comparative genomics. (**A**) Appearance of the colon at necropsy performed for symptoms of extremis. (**B**) Colony morphology of the bacterial isolate obtained from the renal tissue grown aerobically on blood agar. (**C**) Colony morphology of the bacterial isolate grown aerobically on MacConkey agar. (**D**) Average nucleotide identity between AW001 and other *E. coli* genomes of different strains within the B2 phylogroup; K-12 was included as an outgroup strain. (**E and F**) Genomic circle plot comparisons between *E. coli* NC101 and AW001. (**E**) NC101 is shown as a circular genome with blue ORFs along the + and − strand. The green bar represents the AW001 genome compared by nucleotide homology to NC101. Gaps in green show areas of non-homology. (**F**) AW001 is the reference genome in blue, and NC101 is compared by nucleotide homology in green. The legend is underneath each circle plot.

Given the unexpected pathogenicity of non-CRC-1 human microbiota in the CRC mouse model, breeder pairs containing it were closely monitored. One mouse in standard breeding conditions acutely developed signs of illness, prompting necropsy. This mouse had been held at steady state post-inoculation with the non-CRC-1 microbiota and was not undergoing any treatments. Upon necropsy, its colon was markedly dilated, and both kidneys were red and enlarged (Fig. 2A). The appearance of the kidneys suggested that the animal might have had pyelonephritis or a related infection. To assess this possibility, kidney tissues were homogenized, plated on LB agar plates, and incubated aerobically at 37°C. After an overnight incubation, the LB plates contained numerous bacterial colonies that were homogenous in appearance. A single isolate recovered from one of these colonies was sub-cultured onto blood and MacConkey agar for phenotypic characterization. The isolate grew well on both LB and MacConkey media and exhibited moderate alpha-hemolysis on blood agar (Fig. 2B). The isolate grown on MacConkey agar resulted in brilliant pink colonies, indicative of lactose fermentation as often observed with enteric Gram-negative bacteria like *E. coli* (Fig. 2C). We named the isolate AW001, with Round lab strain ID JLR.AW001.

Genomic DNA from AW001 was extracted and sequenced, and the genome was subsequently assembled and annotated. The assembled AW001 genome contains 5,342,215 bases, comprising 4,952 predicted genes. Our group previously published this genome in *Microbiology Resource Announcement* (24). Sequence analysis confirmed that AW001 is an *E. coli* strain belonging to the B2 phylogroup, as determined by ClermonTyping (25). The multi-locus sequence type (MLST) of AW001 was defined as “unknown,” with the most closely related sequence types being 12998, 2831, 141, 10315, 14196, 8290, 1993, and 10905. By average nucleotide identity (ANI), AW001 shares the most genomic sequence identity (99.7%) with the adherent invasive *E. coli* (AIEC) reference strain NC101 (26). The next closest relatives were CTF073, UT189, and APEC 01, which are well-studied reference strains belonging to a large group of pathogens known as extraintestinal pathogenic *E. coli* (ExPEC) (Fig. 2D). ExPEC typically persists within the gastrointestinal tract but has the capacity outside of the gut to cause a number of serious diseases, including urinary tract and bloodstream infections, meningitis, and sepsis (27).

Because AW001 was most similar by genomic sequence identity to NC101, subsequent analyses focused on the similarities and differences between these two strains. To do this, the AW001 genome, which comprised many contigs, was aligned with the complete and assembled NC101 genome. The two genomes aligned almost completely with one another, with only minor gaps, as depicted by breaks in the green sections of the maps depicted in Fig. 2E and F. The blue arrows indicate open reading frames encoded by the plus and minus strands of the NC101 genome. Several prophage regions were identified on the AW001 genome (green and pink sections in Fig. 2E and F), and at least one showed no homology to NC101.

The AW001 genome was further interrogated for antibiotic resistance genes and virulence factors. Several genes associated with antimicrobial resistance were identified, including: (i) a *glpT* variant that encodes a permease with an E448K point mutation that renders bacteria less permeable to the antibiotic fosfomycin (28); (ii) *marR*, which codes for a transcriptional regulator that controls expression of multiple factors that can confer resistance to ampicillin, quinolones, tetracycline, and other antibiotics; (iii) the *pmrB* gene, which is associated with colistin and polymyxin B resistance (29); (iv) *acrF*, which encodes an efflux pump component (30); and (v) the beta-lactamase gene *blaEC* (31) (Table 2). Taken together, AW001 is likely resistant to many different classes of clinically relevant antibiotics.

**TABLE 2:** Key AMR and virulence factors identified in the AW001 *E. coli* genome compared with NC101^*a*^

Gene(s)	Protein ID	Type	Protein name and function	AW001	NC101	Location
*acrF*	WP_001273251.1	AMR	AcrF multidrug efflux RND transporter permease	Present	Present	Chromosome
*blaEC*	WP_001556381.1	AMR	BlaEC family class C beta-lactamase	Present	Present	Chromosome
*glpT*	WP_000948732.1	AMR	GlpT fosfomycin resistant	Present	Present	Chromosome
*marR*	WP_000799375.1	AMR	MarR multidrug resistant	Present	Present	Chromosome
*pmrB*	WP_001052123.1	AMR	PmrB colistin resistant	Present	Present	Chromosome
*traB,C,P*	WP_021553196.1	Conjugation	F conjugative plasmid, F pilin operon	Present	**Absent**	Chromosome
*druA,B*	WP_000548583.1	Defense	DruA Druantia type 1 anti-viral defense	Present	**Absent**	Chromosome
*idnR,T*	WP_001560766.1	Metabolism	IdnR L-idonate catabolism operon	Present	**Absent**	Chromosome
*pduT,P*	WFA96297.1	Metabolism	Pdu propanediol-utilization metabolosome	**Absent**	Present	Chromosome
*ariR*	WP_000888771.1	Stress	Biofilm/acid-resistance regulator AriR	Present	Present	Chromosome
*emrE*	WP_001070440.1	Stress	EmrE multidrug efflux SMR transporter	Present	Present	Chromosome
*astA*	WP_000989438.1	Virulence	EAST1 heat-stable enterotoxin	Present	Present	Chromosome
*cbtA*	WP_000854814.1	Virulence	CbtA type IV toxin-antitoxin system	Present	Present	Chromosome
*ccdA*	WP_000125566.1	Virulence	CcdA type II toxin-antitoxin system CcdA	Present	Present	Chromosome
*chuA*	WP_000089583.1	Virulence	ChuA outer membrane hemin receptor	Present	Present	Chromosome
*ClbA*	WP_001217110.1	Virulence	Colibactin synthesis proteins (*pks*)	Present	Present	Chromosome
*entH*	WP_000637953.1	Virulence	EntH proofreading thioesterase	Present	Present	Chromosome
*fdeC*	WP_000092543.1	Virulence	FdeC inverse autotransporter adhesin	Present	Present	Chromosome
*fimH*	WP_000832236.1	Virulence	SfaH fimbrial protein subunit	Present	Present	Chromosome
*fliP*	WP_334615852.1	Virulence	Flagellar type III secretion system	Present	Present	Chromosome
*gspA*	WP_000107592.1	Virulence	GspA, C, D etc. type II secretion system	Present	Present	Chromosome
*gspG*	WP_001087296.1	Virulence	GspG type II secretion system major pseudopilin	Present	Present	Chromosome
*hcp*	WP_000458845.1	Virulence	Hcp family type VI secretion system effector	Present	Present	Chromosome
*hecB*	WP_334616364.1	Virulence	HecB family hemolysin secretion/activation protein	Present	Present	Chromosome
*hhA*	WP_001333231.1	Virulence	Hha hemolysin expression modulator	Present	Present	Chromosome
*ibsE*	WP_001387082.1	Virulence	Ibs family toxin type I toxin-antitoxin system	Present	Present	Chromosome
*Irp1,2*	WP_000369530.1	Virulence	Yersiniabactin siderophore	Present	Present	Chromosome
*iss*	WP_001298464.1	Virulence	Iss increased serum survival lipoprotein	Present	Present	Chromosome
*ldrD*	WP_001295224.1	Virulence	Ldr family protein type I toxin-antitoxin system	Present	Present	Chromosome
*mchB*	WP_001375214.1	Virulence	H47 microcin	Present	**Absent**	Chromosome
*mchF*	WP_001518504.1	Virulence	MchF microcin H47 export transporter peptidase	Present	**Absent**	Chromosome
*neuC*	WP_000723250.1	Virulence	Polysialic acid biosynthesis protein P7	Present	Present	Chromosome
*ompA*	WP_001518466.1	Virulence	Outer membrane protein	Present	Present	Chromosome
*paeA*	WP_000935036.1	Virulence	Hemolysin family protein	Present	Present	Chromosome
*papA,H*	WP_000920486.1	Virulence	PapA pilli operon	Present	**Absent**	Chromosome
*sitC*	WP_001101732.1	Virulence	MntB manganese transport membrane protein	Present	Present	Chromosome
*sslE*	WP_001034565.1	Virulence	SslE lipoprotein metalloprotease	Present	Present	Chromosome
*TssE*	WP_000106967.1	Virulence	TssE type VI secretion system baseplate subunit	Present	Present	Chromosome
*tssJ*	WP_000484008.1	Virulence	TssJ type VI secretion system lipoprotein	Present	Present	Chromosome
*vgrG*	WP_001350146.1	Virulence	VgrG type VI secretion system tip protein	Present	Present	Chromosome
*ybtE*	WP_001518699.1	Virulence	2,3-Dihydroxybenzoate-AMP ligase	Present	Present	Chromosome
*ybtP*	WP_001327262.1	Virulence	YbtP yersiniabactin ABC transporter	Present	Present	Chromosome
*ybtQ*	WP_001295637.1	Virulence	YbtQ yersiniabactin ABC transporter	Present	Present	Chromosome
*yqfA*	WP_000250274.1	Virulence	Hemolysin III family protein	Present	Present	Chromosome
*virB4*	WP_000105979.1	Virulence	VirB4 family type IV secretion system	**Absent**	Present	Plasmid
*virB9*	WP_001299943.1	Virulence	VirB9 type IV secretion system protein	**Absent**	Present	Plasmid
*virB11*	WP_000017224.1	Virulence	P-type DNA transfer ATPase VirB11	**Absent**	Present	Plasmid
*unnamed*	WP_001328552.1	Virulence	Cag pathogenicity island Cag12 family protein	**Absent**	Present	Plasmid

^*a*^ Boldface indicates the places where the two genomes differ.

AW001 also encoded multiple virulence factors known to be associated with AIEC, ExPEC, and other pathogenic strains of *E. coli* (24, 32). Interestingly, like NC101 and many ExPEC isolates, AW001 carries the *pks* gene cluster used for the production of the DNA-damaging toxin colibactin, which could help explain the massively enhanced tumorigenesis observed in in mice harboring the non-CRC-1 microbiota in the AOM/DSS CRC model (see Fig. 1F and G). Other virulence-associated genes encoded by AW001 include the heat-stable enterotoxin AstA, the antimicrobial microcin H47, and α-hemolysin. AW001 also encodes a putative type II secretion system, a type III secretion system, and a type VI secretion system with multiple putative effectors, all of which may enhance the virulence potential of *E. coli* pathogens (12, 33). Of note, AW001 did not encode cytotoxic necrotizing factor, cytolethal distending toxin, or cycle-inhibiting factor, which are often used by *E. coli* pathogens to obstruct the host cell cycle and induce megalocytosis (Table 2) (34). Like many *E. coli* strains, AW001 also carried genes for type I, II, and IV toxin-antitoxin systems, which may contribute to bacterial stress resistance and persistence phenotypes (35, 36).

Interestingly, most of the known and putative virulence factors found in AW001 are also seen in the AIEC isolate NC101, with a few notable exceptions. For example, AW001, but not NC101, possesses the *mchB* and *mchF* genes that code for the H47 microcin and its export transporter peptidase, respectively. This microcin can act as an antibacterial peptide that may be used to help *E. coli* better compete with other microbes (37, 38). *E. coli* microcins can cross the gut blood barrier, and some can act as genotoxins to eukaryotic host cells or exacerbate colitis (39, 40). Another major difference between AW001 and NC101 is that the latter has a plasmid that encodes elements of a type IV secretion system (T4SS), including the *virB4*, *virb9*, and *virb11* genes, encompassed within a region with similarities to the T4SS and Cag pathogenicity island carried by many proteobacteria, including *Helicobacter pylori* (Table 2) (41). While our sequencing analysis of AW001 suggests that this microbe may also carry a plasmid, AW001 does not encode a T4SS or a Cag pathogenicity island. Other notable differences between NC101 and AW001 include the presence of the F conjugative plasmid in AW001 and F pili operon, which is absent in NC101. Perhaps the most disease-relevant difference between NC101 and AW001 is the presence in AW001 of the *pap* operon, which encodes P pili (Table 2). These filamentous adhesive organelles can mediate bacterial adherence to host glycolipid receptors within the kidneys, promoting the development of pyelonephritis (42). The expression of P pili by AW001 could help explain how the kidneys of the mouse colonized with AW001 were infected.

### *E. coli* AW001 exacerbates acute colitis and is lethal in a mouse model of sepsis

**Fig 3:**
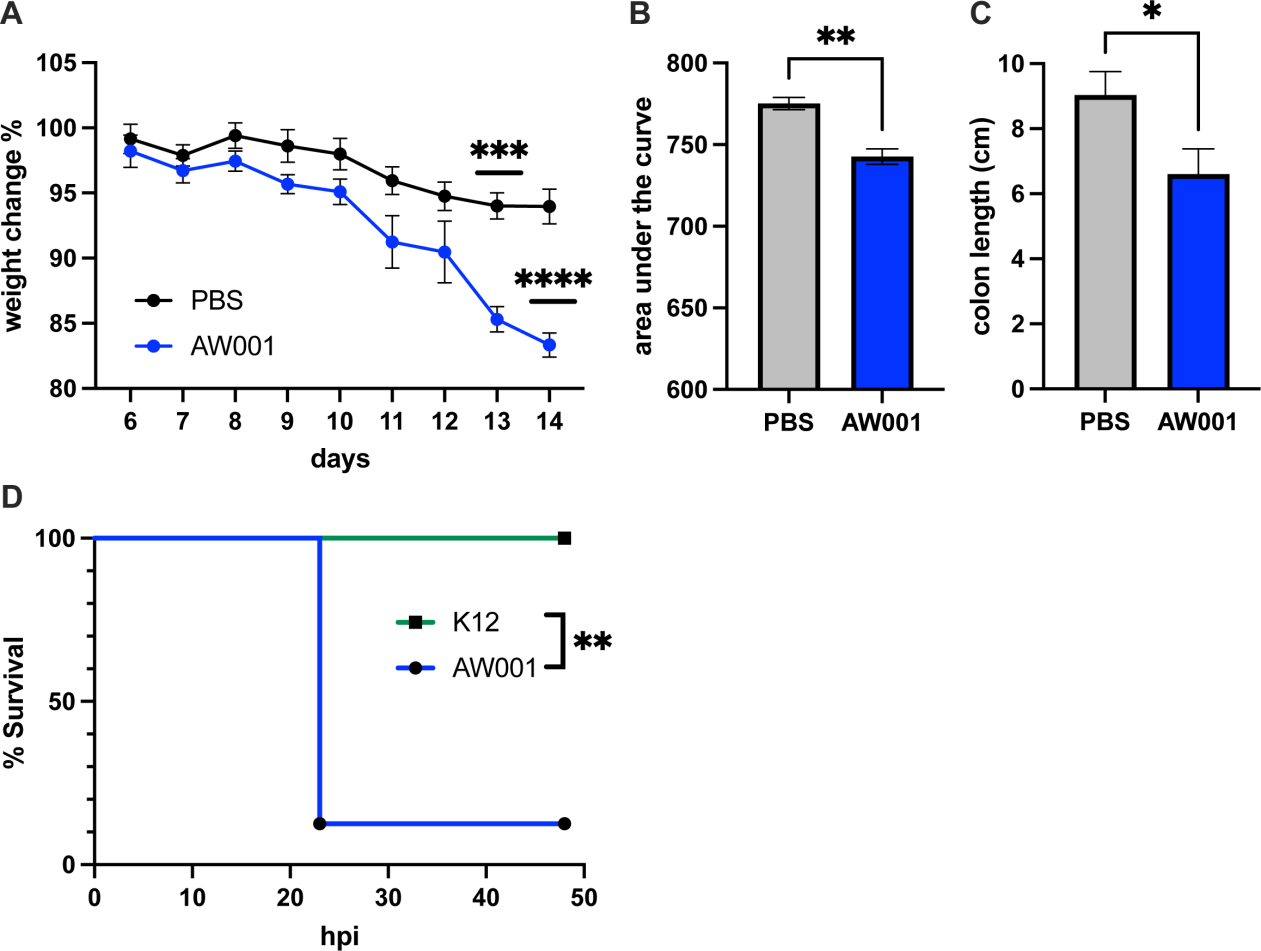
*E. coli* AW001 exacerbated colitis and was lethal in a mouse model of sepsis. (**A**) Mouse weight change during acute DSS treatment after oral administration of *E. coli* AW001 vs PBS control. (**B**) Area under the curve of the weight change. (**C**) Colon length at sacrifice comparing mice colonized with AW001 or PBS control. *N* = 6 males, split between groups. (**D**) Kaplan–Meier survival plot of mice following i.p. inoculations with either AW001 or the *E. coli* K12 strain MG1655. *P* = 0.0018, by log-rank Mantel–Cox test for AW001 versus MG1655. *n* = 7 animals injected with AW001 and 6 with K12, split about evenly between sexes. The one surviving mouse from the AW001 group was female. Differences were analyzed using unpaired two-tailed *t*-tests: ns, *P* ≥ 0.05; *, *P* ≤ 0.05; **, *P* ≤ 0.005; ***, *P* ≤ 0.0005; ****, *P* ≤ 0.0001. Animal weight change analyzed by one-way ANOVA with multiple comparisons. Error bars are ± SEM.

Because the microbiota from which AW001 was isolated led to massive tumors and large-scale mouse death in AOM/DSS-treated mice, often at an early stage prior to the onset of tumorigenesis (see Fig. 1), we wondered whether the isolate AW001 alone was sufficient to induce colitis in an acute colitis mouse model. To test this possibility, male Swiss Webster mice devoid of any endogenous *E. coli* were inoculated with AW001 for 5 consecutive days, while control mice received PBS. After 1 day of rest, the mice were given DSS in their drinking water, and their weight was monitored for the next 8 days (days 6–14). At the end timepoint, mice were euthanized, and their colons were measured. Mice colonized with AW001 lost significantly more weight than the PBS controls (Fig. 3A and B), and their colons were significantly shorter (Fig. 3C), indicative of more severe disease. When organs were plated to quantify numbers of disseminated *E. coli*, the mice given AW001 showed bacteria present within the kidneys, liver, and spleen (Fig. S1A through C). These bacteria appeared to be *E. coli* based on growth characteristics on MacConkey agar. No bacteria were recovered from tissues taken from the PBS control group (Fig. S1A through C).

To further investigate the pathogenic potential of AW001, we tested *E. coli* AW001 in a mouse model of sepsis, as previously described (43). Briefly, C57Bl/6 mice were inoculated via intraperitoneal injections with either AW001 or with a non-pathogenic *E. coli* K12 strain, and animals were closely monitored for 48 h for subsequent signs of morbidity and mortality. After 23 h, all of the AW001-inoculated mice met mortality endpoints, except for one female (Fig. 3D). All of the mice inoculated with the K12 strain survived, despite the fact that this strain has 96.8 genomic similarity to AW001. Similar results were obtained using Swiss Webster mice, as employed above in the AOM/DSS CRC model (Fig. S1D). Taken together, our data indicate that AW001 can act as a particularly lethal extraintestinal pathogen in mice.

## DISCUSSION

Control microbiotas from healthy donors have long been used as controls in microbiology and microbiome studies. However, at present, our understanding of what makes a microbiota “healthy” or “unhealthy” is limited and highly context-dependent, and we still cannot conclusively state if one microbiota will lead to disease or not without testing. This study provides a remarkable example of a non-disease human microbiota harboring a potentially pro-tumorigenic and potentially deadly opportunistic pathogen, highlighting the pathogenic potential of seemingly benign microbial communities. It also indicates that there may be a third microbiota type, that of “apparently healthy.” In this definition, despite a person being asymptomatic, an unknown history of dysbiosis or antibiotics use may have led to the presence or abundance of pro-tumorigenic microbial species. This urges caution and the need for human FMT screening prior to usage clinically or experimentally.

As for the newly discovered human *E. coli* AW001 isolate, it remains unclear if the apparent pyelonephritis that occurred in the mouse from which it was isolated was a result of hematogenous dissemination or ascending urinary tract infection. The former possibility seems more likely, given that mice generally do not naturally develop ascending UTI (44). The organism that is most similar genomically to AW001 by ANI calculations is the well-characterized adhesive-invasive *E. coli* (AIEC) isolate NC101. Like NC101, AW001 carries the *pks* gene cluster, which codes for the pro-tumorigenic genotoxin colibactin. Expression of colibactin by AW001 may be linked to the ability of the non-CRC-1 human microbiota to promote tumorigenesis in our murine CRC model. The high level of genetic similarity between AW001 and NC101, including the presence of *pks* genes in both isolates, as well as the ability of AW001 to exacerbate disease phenotypes in a CRC model, suggests that AW001 is an AIEC strain. However, AW001 and NC101 are distinct in multiple ways, including their source (NC101 is mouse-derived while AW001 is from a human patient), their sequence types, and their overall repertoire of virulence genes and other factors. Several of the genes unique to AW001 (relative to NC101) likely enable AW001 to disseminate from the gut into the kidneys and other organs, eliciting pyelonephritic and sepsis-like disease. The capacity of AW001 to spread systemically, possibly via breaks in the intestinal barrier that can develop within the inflamed gut, is reminiscent of ExPEC strains. More specifically, the capacity of AW001 to colonize the kidneys and elicit pyelonephritis-like disease is characteristic of a subset of ExPEC strains known as uropathogenic *E. coli* (UPEC) (42, 45). The tropism of AW001 for the kidneys may be facilitated by the expression of P pili, the genes for which are absent in NC101. In total, these observations suggest that AW001 is a hybrid strain, possessing key genotypic and phenotypic characteristics of both AIEC and ExPEC strains.

Thus, we present a novel human-derived isolate that adds to the scientific knowledge of *E. coli* strains, particularly those that harbor colibactin and other elements that can cause inflammation and DNA damage, such as microcins, and are associated with tumorigenesis. In this study, we did not test if AW001 by itself was sufficient to promote colon tumorigenesis; however, the microbiota from which AW001 was isolated was clearly associated with increased tumor numbers and sizes, as well as greater lethality during the AOM/DSS model (as shown in Fig. 1). We did show that AW001 exacerbated colitis in an acute model of colitis and led to complete lethality in a sepsis model, leading to the conclusions that AW001 was likely responsible for the colitis-associated tumorigenesis and was the reason for the original mouse death from which it was isolated from the kidney. Taken together, we present a novel human-derived, potentially pro-tumorigenic and pro-colitogenic *E. coli* with invasive potential in mice. Further investigation is needed to better understand correlates of pathogenicity for strains like AW001 in humans, given that AW001 was isolated from a human patient without colon cancer or other known illnesses.

## MATERIALS AND METHODS

### Collecting and processing of human fecal samples

Fecal samples were provided by Dr. Ulrich’s research group and the ColoCare Study (ClinicalTrials.gov NCT02328677), an international, multi-site cohort of newly diagnosed stage I–IV colorectal cancer patients (ICD-10 C18-C20) (17). The ColoCare Study design has previously been described (17, 18, 46). All analyses in this paper are based on data collected from patients with stage I–IV colorectal cancer enrolled between October 2010 and March 2018 at study sites at the National Center for Tumor Diseases and University of Heidelberg (Heidelberg, Germany) and the Huntsman Cancer Institute (HCI) (Utah, USA), with available stool samples. The study was approved by the institutional review boards of the respective institutions, and all patients provided written informed consent. Stool samples were collected by patients prior to surgery and immediately frozen and stored at −80°C. If patients received neoadjuvant treatment, stool samples were collected at least 2 weeks after completion of treatment. Standardized biospecimen collection questionnaires were used to collect specific quality control data and covariates, including the date and time of stool specimen collection and prior use of antibiotics and NSAIDs (17, 47).

### Mouse colonization with human microbiotas

Individuals were chosen based on CRC status and whether age- and sex-matched controls were available. All human CRC patients had not taken any antibiotics for at least 3 months prior to the sample. Fecal samples were all stored at −80*°*C until processing and then refrozen at −80*°*C post-colonization. Colonization of mice was performed as previously described by Goodman and colleagues (19) and commonly done in our lab (20). Briefly, fecal samples were allowed to thaw at room temperature and resuspended in reduced PBS at 15 g/mL^−1^. Samples were vortexed for 5 min and then incubated at room temperature for 5 min to allow solid particles to settle at the bottom of 15 mL conical tubes. Two hundred microliter aliquots of the suspended liquid fractions were then gavaged into germ-free (GF) mice 3 weeks prior to the start of the experiment. Mice were removed from gnotobiotic isolators and immediately inoculated with human microbiotas. GF Swiss Webster mice, initially purchased from Taconic but maintained by the UofU Gnotobiotics Core, were utilized for all human microbiota engraftment experiments and, as an outbred strain, for their potential genetic diversity as a model for human disease in the CRC model. Mice were then housed in cages with HEPA filters segregated by microbiota.

### Bacterial isolation

*E. coli* AW001 was recovered from the kidneys of an ex-germ-free Swiss Webster mouse that had recently undergone a human-to-mouse FMT 3 weeks prior. Kidney tissue was homogenized and plated on LB agar plates and incubated aerobically at 37°C. The plated kidney homogenate yielded colonies that were too numerous to count, all with the same colony morphology on LB agar. Growth from the primary plate was subcultured to single colonies. These were further subcultured onto blood agar and MacConkey agar for phenotypic characterization.

### AOM/DSS colon tumorigenesis model

All mice were given 10 mg/kg azoxymethane (AOM) by intraperitoneal injection on day 0 of the model. Swiss Webster mice were provided 2% (wt/vol) dextran sulfate sodium (DSS) (MP Biomedicals, Cat #0216011090) in the drinking water *ad libitum* at three intervals for 5 days and were sacrificed on day 70. Animal weights were obtained before AOM injection and DSS treatment, and mice were monitored for weight change throughout the experiments. These models have been previously described and performed in our lab (16, 48).

### Colitis mouse model

Six-week-old male Swiss Webster mice that were devoid of any background *E. coli* were inoculated by oral gavage with 10^7^ CFU of the *E. coli* strain AW001 or PBS for 4 consecutive days. On day 6, 2.5% DSS (MP Biomedicals, Cat #0216011090) was administered in the drinking water of all mice, and mice were weighed daily throughout the model.

### Sepsis mouse model

Adult male and female C57BL/6 mice and Swiss Webster mice were injected intraperitoneally with 200 mL of PBS containing about 1 × 10^8^ CFU of either AW001 or the control K12 strain MG1655, as previously described (43). Mice were then monitored for 48 h for signs of illness and morbidity.

### Primers used to assay bacterial toxins in human and mouse microbiotas

Previously published primer sequences and PCR conditions for bacteria and their toxins were used in this study. *E. coli* PKS toxin genes *clbA*, *clbP*, and *clbB* were analyzed in this study. *clbA* F and R primers: *clbA*_F: 5′-CAGATACACAGATACCATTCA-3′; *clbA*_R: 5′-CTAGATTATCCGTGGCGATTC-3′. *clbP* F and R primers: *clbP*_F: 5′-GTGAACTGAGCGAAATATTGGCTAATC-3′; *clbP*_R: 5′-TTACTCATCGTCCCACTCCTTGTTG-3′ were used as described (22). *clbB* F and R primers: clbB_F: 5′-GCAACATACTCGCCCAGCT-3′; clbB_R: 5′-TCTCAAGGCGTTGTTGTTTG-3′ as published (13). Primer sequences for the ETBF toxin gene *bft* used were bft_F: 5′-GCGAACTCGGTTTATGCAGT-3′; bft_R: 5′-GTTGTAGACATCCCACTGGC-3′, as published (13). An additional set of *bft* primers was also used: Bft_F 5′-TGGGAGATGAGTTCGCAGTATTA-3′; Bft_R 5′-CCAACCGAGATTTTTAGCGATTAT-3′ (49). Primer sequences used for *F. nucleatum* identification (All-F6): 5′-CGGGAGGCAGCAGTGGGGAAT-3′; (Fn-R6): 5′-TTGCTTGGGCGCTGAGGTTC-3′, and PCR conditions were used exactly as published (23).

### Genome sequencing

*E. coli* AW001 was sequenced as previously published (24). Briefly, DNA was extracted from a pure culture of *E. coli* AW001 in the Mulvey lab using the Qiagen DNA isolation kit (Invitrogen) and was sequenced using an Illumina NovaSEQ6000 instrument through the University of Colorado Anschutz Genomics and Microarray Core, generating 151 bp paired-end reads. Adapters and phiX sequences were trimmed using BBDuk v37.10, and reads were trimmed based on quality scores using seq-qc 2.0.2 and assessed for quality using FastQC v0.11.9 (50).

### Genome assembly and annotation

Sequences were assembled *de novo* using Unicycler through PATRIC (51–53). The assembled genome was annotated using Genbank PGAP v6.6 (54, 55). Quality estimation was performed using CheckM (56).

### Genome analysis

Phylogroup was predicted by using ClermonTyping (v23.06) (25). MLST was determined using the Center for Genomic Epidemiology web tool (MLST 2.0 Server). We used AMRFinderPlus to analyze the genome for antimicrobial resistance (57). AMRFinderPlus, BLAST+, and targeted searching through annotations were used to analyze the AW001 genome for virulence factors known to be associated with pathogenic strains of *E. coli* (32). FastANI v0.1.3 was used for ANI calculations and graphics (58). Proksee was used for circular mapped genome comparisons between AW001 and NC101 (59). Within Proksee, both Prokka and Genbank PGAP annotations were both used to compare genomes, and PHASTEST was used to annotate phage elements (55, 60).

### Statistical analysis

Figure creation and statistical analysis were performed with Prism 10 software. Specific statistical tests are indicated in figure legends. Statistical analyses for sequencing experiments are indicated in the figures and in the relevant Materials and Methods section. Biorender was used for figure schematics.

## Data Availability

The genome can be found under the Genbank accession JAYWIW000000000 *Escherichia coli* AW001, Biosample SAMN39433337, and is part of Bioproject PRJNA1064449. The raw reads are linked and have been uploaded into the SRA. All other data needed to evaluate the conclusions in the paper are available within the main text or supplemental materials.

## SUPPLEMENTAL MATERIAL

Figure S1 msphere.00219-26-s0001.pdf *E. coli* AW001 dissemination.

Legend msphere.00219-26-s0002.docx Figure S1 legend.

Table S1 msphere.00219-26-s0003.docx PCR of *E. coli* and colibactin genes.

ASM does not own the copyrights to Supplemental Material that may be linked to, or accessed through, an article. The authors have granted ASM a non-exclusive, world-wide license to publish the Supplemental Material files. Please contact the corresponding author directly for reuse.
