# Supplementary figures and images for: Isolation of a highly virulent colibactin-positive tumor-promoting strain of Escherichia coli from the gut microbiota of an adult
Source: mSphere. 2026 May 7;11(5):e00219-26. doi: 10.1128/msphere.00219-26 (PMC13203963; doi:10.1128/msphere.00219-26)

**A**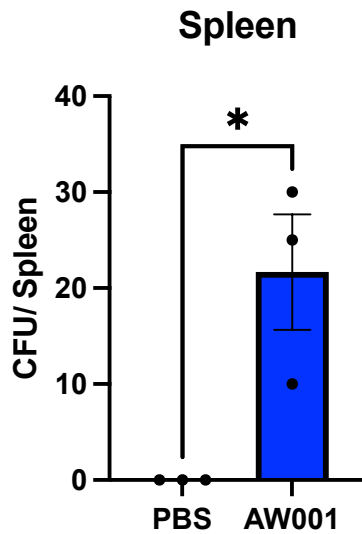**B**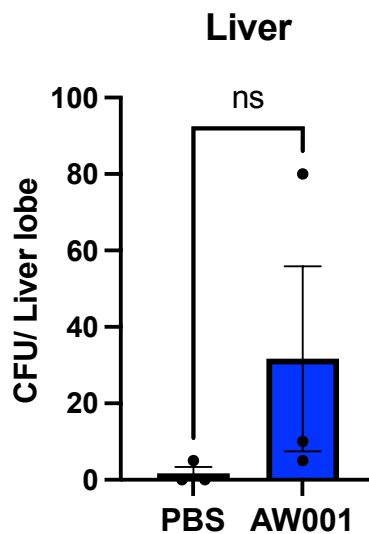**C**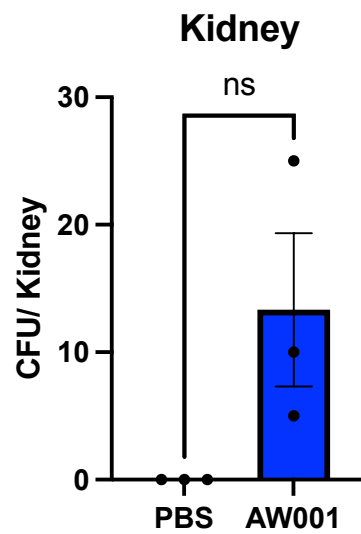**D**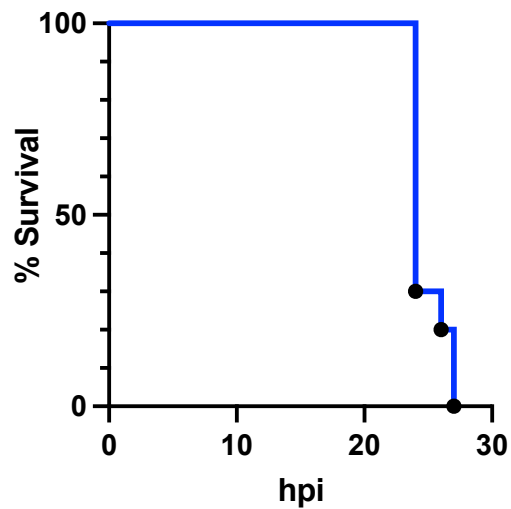

Supplement: Figure S1 — E. coli AW001 dissemination. [file msphere.00219-26-s0001.pdf]
