# Supplementary material for: Isolation of a highly virulent colibactin-positive tumor-promoting strain of Escherichia coli from the gut microbiota of an adult
Source: mSphere. 2026 May 7;11(5):e00219-26. doi: 10.1128/msphere.00219-26 (PMC13203963; doi:10.1128/msphere.00219-26)
Supplement: Legend — Figure S1 legend. [file msphere.00219-26-s0002.docx]

**Supplemental Figure 1, related to Figure 3:**

*E. coli* AW001 dissemination to kidney **(A)**, liver **(B)**, and spleen **(C)** after acute colitis model shown in Fig. 3A-C. Differences were analyzed using unpaired two-tailed t tests where ns = >0.05, * p = <0.05, **p = <0.005 *** p = <0.0005, ****p = <0.0001. Error bars are +/- SEM.
