# Supplementary material for: Isolation of a highly virulent colibactin-positive tumor-promoting strain of Escherichia coli from the gut microbiota of an adult
Source: mSphere. 2026 May 7;11(5):e00219-26. doi: 10.1128/msphere.00219-26 (PMC13203963; doi:10.1128/msphere.00219-26)
Supplement: Table S1 — PCR of E. coli and colibactin genes. [file msphere.00219-26-s0003.docx]

| Supplemental Table 1: PCR of *E. coli* and colibactin genes from non-CRC-1 mouse fecal samples | | | | | | |
| --- | --- | --- | --- | --- | --- | --- |
|  |  |  |  |  |  |  |
| Microbiota | Bacteria 16S rDNA | colibactin toxin genes | |  |  |  |
| non-CRC-1 | *E. coli*+ | *clbA* + | *clbP* + |  |  |  |
|  |  |  |  |  |  |  |
|  |  |  |  |  |  |  |
|  |  |  |  |  |  |  |
|  |  |  |  |  |  |  |
|  |  |  |  |  |  |  |
